# What Term to Choose: Ambiguous Genitalia or Disorders of Sex Development (DSD)?

**DOI:** 10.3389/fped.2019.00316

**Published:** 2019-07-30

**Authors:** Smail Acimi

**Affiliations:** Department of Visceral Surgery, Children's Hospital Canastel, University of Oran, Oran, Algeria

**Keywords:** ambiguous genitalia, disorders of sex development, DSD, feminizing genitoplasty, masculinized genitoplasty, Chicago consensus

The meeting of Chicago, in 2005, which regrouped about 50 experts from 10 countries marked a milestone in the history of genital malformations. However, the two main changes proposed by the Chicago consensus (1), concerned the terminology and the timing of the surgical correction deserves a discussion.

1- The changes in terminology: it is clear that “Terms such as intersex, pseudohermaphroditism, hermaphroditism, sex reversal are perceived as potentially pejorative by patients” (1). However, I think that the term “ambiguous genitalia,” widely used in the past is not as pejorative, and this term was in particular more precise in its definition, with clear limits.

We talk about ambiguous genitalia when the appearance of the external genitalia of a new-born, does not resemble that of a girl, nor a boy, but rather a form between the two. However, this definition remains incomplete, and ambiguous genitalia must also include patients with phenotypes that do not correspond to the genetic sex, a discordance between genital appearance and karyotype. Usually, these cases are discovered later in older children and at puberty (2).

Whereas, the definition and limits of the term disorders of sex development (DSD) remains fuzzy, this term refers to a wider range of pathologies completely different by origin, pathogenesis, clinical expression, and their therapeutic method (ex: ambiguous genitalia by congenital adrenal hyperplasia and 46, XY cloacal exstrophy). Thus, some questions remain unanswered, for example; Why include 46, XY cloacal exstrophy and not 46XX bladder exstrophy (with two hemiclitoris'), Why include aphallia and not diphallia…?

I agree with González and Ludwikowski who reports that “Although the term DSD was widely accepted by patients, families and health professionals, it created a new series of problems” (3). In Turkey, a Muslim country, only 2% of parents of DSD patients preferred using the term DSD (4). A nomenclature which remains controversial (5).

I think there are two elements that greatly influence the therapeutic approach: the karyotype and the causes of ambiguous genitalia. Thus, they must be mentioned in the terminology and ambiguous genitalia can be classified into four groups: enzymatic deficiencies, abnormalities in the androgen receptor, abnormalities of gonad development, and maternal androgens excess.

a)- Enzymatic deficiencies
  -of glandular origins (testis and adrenal): such as
  46XY/17β-hydroxystéroide-déshydrogénase(17 β-HSD3) deficiency
  46,XX/ congenital adrenal hyperplasia (CAH)
  46XY/StAR deficiency
  46XY/3β-hydroxysteroid dehydrogenase (3β-HSD) deficiency
  -of peripheral origins: such as 46XY/5-alpha reductase 2 (5-AR2) deficiency
b)- Abnormalities in the androgen receptor: such as
  46XY/Complete Androgen Insensitivity Syndrome (CAIS)
  46XY/Partial Androgen Insensitivity Syndrome (PAIS)
c)- Abnormalities of gonad development: such as
  46,XX or 46,XX/XY or 46,XY/Ovo-testis (historically called true hermaphroditism)
  45X/46 XY/Mixed gonadal dysgenesis
  46XY/pure gonadal dysgenesis.
  46XY/Leydig cell hypoplasia type1 and 2
  45X/Turner syndrome
  47XXY/ Klinefelter syndrome
d)- Maternal androgens excess (very rare)
  46XX/Virilized by maternal tumor
  46XX/Virilized by exogenous androgens

2- Timing of surgical correction: one of the main recommendations of the Chicago consensus was to delay vaginoplasty at adolescence, “when the patient is psychologically motivated and a full partner in the procedure” (1).

There is a surgical reason to delay the vaginoplasty at adolescence, many patients who undergo feminizing genitoplasty in early childhood require surgical correction at puberty, but there is also an innuendo that it is necessary to wait until the patient can participate in the choice of his sex. However, if this is normal or tolerated in the Western world, with a population that represents only about 18% of the world's population, in most Muslim countries, the religious factor plays an important role in the social life of the people, and it is difficult to change the identity of the gender at adolescence, and very difficult, even impossible to do it at the adulthood.

In some western countries, intersex activists have encouraged legislative bodies to ban genital surgery without the individual's informed consent (5). While DSD patients and their parents were not consulted.

Mouriquand et al. reported recently that “Ten years after the Chicago consensus meeting, genital surgery continues to raise questions and criticisms…There is no consensus regarding the indications, the timing, the procedure, and the evaluation of outcome of DSD surgery” (6).

Patients who require vaginoplasty could be divided into two groups (2):

-Patients with vaginal agenesis: such as the complete androgen insensitivity syndrome (CAIS) and some cases of 46, XY DSD, such as Leydig cell hypoplasia type1. These forms, often manifest later, in older children or at puberty, through clinical signs, such as inguinal hernia; virilization or the delayed of primary amenorrhea in a girl; and breast development or apparition of cyclical hematuria in a boy. Thus, the timing of vaginoplasty does not arise and the first recommended treatment of vaginal agenesis at the adolescence is the vaginal dilatation.

-Patients with persistent Müllerian duct: such as 46, XX DSD, Ovotestis DSD, and mixed gonadal dysgenesis are often discovered at birth, they are characterized as having ambiguous genitalia with the presence of a uterus, fallopian tubes, and an upper vagina. The aim of the vaginoplasty is to separate the openings of the vagina and urethra, and to connect the vaginal cul-de-sac to perineal skin.

Early assignment of an appropriate sex is an important step for a good development of gender identity. The current trend is to keep DSD individuals with Y material in the male gender despite the unlikelihood of fertility and an uncertain surgical outcome (6). When the male gender is chosen, things are simpler and the masculinized genitoplasty must be accomplished in early infancy, definitely before the age of 10. During puberty and adulthood, hyper vascularization of the penis which accompanies the hypersecretion of androgens makes the surgical repair more hemorrhagic, and more difficult.

However, in some cases of ambiguous genitalia, choosing the sex gender remains a complex and difficult step, particularly for ovotestis DSD and gonadal dysgenesis with 46,XYor 45,X/46,XY karyotype. Nevertheless, in two forms of ambiguous genitalia, 17 β -hydroxysteroid dehydrogenase (17β-HSD3) deficiency and 5 α-Reductase 2 deficiency, the feminizing genitoplasty must never be performed during childhood. These cases virilize and often change the sexual behavior and gender identity at puberty.

Facing high rates of poor cosmetic results and difficulties in sexual intercourse, reported in women who underwent feminizing genitoplasty for ambiguous genitalia during their childhood, surgeons were the main target of critics at the meeting in Chicago. However, the surgical repair of these urogenital malformations has been considerably improved over the last 20 years. Several cohorts of adult patients who underwent a feminization procedure at various ages have recently been interviewed in different French hospitals, all claimed that early surgery is highly preferable to late surgery (6). Nevertheless, as mentioned at this meeting, it is imperative that these patients be treated in specialized centers where at least 50 surgical repairs of ambiguous genitalia are performed per year.

I think that when the diagnosis of the type of ambiguous genitalia is accurately made, and the feminizing genitoplasty is proposed, which is not always the case, the vaginoplasty should be performed as early as possible, during early infancy, at the same time with clitoroplasty and creating labia minora, preferably between 6 and 24 months (7). It is difficult to accept the sentence reported at the Chicago consensus “Emphasis is on functional outcome rather than a strictly cosmetic appearance” (1). I think that the cosmetic result should be as important as the functional result.

The cosmetic result depends on three important criteria (2):

- The glans should be small, and its apparent part should not exceed 5 mm.
- Presence of the labia minora and its appearance.
- The nature of the tissue used in covering the area located between the two labia minora. This area should be covered by the wall of the common urogenital canal, never by perineal skin.

## Author Contributions

The author confirms being the sole contributor of this work and has approved it for publication.

### Conflict of Interest Statement

The author declares that the research was conducted in the absence of any commercial or financial relationships that could be construed as a potential conflict of interest.
